# The Prevalence and Underreporting of Needlestick Injuries among Dental Healthcare Workers in Pakistan: A Systematic Review

**DOI:** 10.1155/2018/9609038

**Published:** 2018-02-12

**Authors:** Mehak Pervaiz, Ruth Gilbert, Nasreen Ali

**Affiliations:** ^1^APPNA Institute of Public Health, Jinnah Sindh Medical University, Karachi, Pakistan; ^2^School of Healthcare Practice, University of Bedfordshire, Putteridge Bury, Luton, Bedfordshire LU2 8LE, UK; ^3^Institute of Health Research, University of Bedfordshire, Putteridge Bury, Luton, Bedfordshire LU2 8LE, UK

## Abstract

Needlestick injuries (NSIs) are a major occupational health problem among dental healthcare workers (HCWs) in Pakistan, which places them at a significant risk of acquiring blood-borne infections. However, not all NSIs are reported, leading to an underestimation of the actual prevalence. The harmful impacts of NSIs on the healthcare delivery necessitate an urgent need to measure its actual prevalence. *Objectives*. The aim of this study was to review literature to estimate the prevalence and reporting rates of NSIs among dental-HCWs in Pakistan. *Methods*. 713 potentially relevant citations were identified by electronic databases and hand searching of articles. Nine primary studies were subsequently identified to be included in the review. *Results*. The results of the included studies indicate that the prevalence of NSIs among Pakistani dental-HCWs was between 30% and 73%. The rate of reporting of NSIs was between 15% and 76%, and the most common reason was found to be the lack of awareness regarding the reporting system, or of the need to report NSIs. *Conclusion*. It is evident from the review of the included studies that there is a significantly high prevalence and a low rate of reporting of NSIs among dental-HCWs in Pakistan, suggesting the need to setup an occupational health department in dental settings, for preventing, managing, recording, and monitoring NSIs.

## 1. Introduction

Globally, an estimated two million healthcare workers (HCWs) experience a needlestick injury (NSI) each year [1] putting them at risk of infectious diseases such as hepatitis B virus (HBV), hepatitis C virus (HCV), and human immunodeficiency virus (HIV) [2, 3]. Globally, more than a third of hepatitis B and hepatitis C cases and approximately 5% of HIV cases result from an NSI [1] despite evidence to show effective infection control policies that can successfully prevent HBV seroconversion and minimise rates of HCV and HIV seroconversion following an NSI [4]. NSIs have also been shown to transmit other bacterial, fungal, or viral infections, including blastomycosis, cryptococcosis, diphtheria, herpes, malaria, mycobacteriosis, and syphilis [5]. It is also reported that in up to 12% of cases, NSIs may also lead to psychiatric morbidity including posttraumatic stress disorder (PTSD) [6]. Furthermore, the presence of blood-contaminated saliva increases the risk of infection with blood-borne viruses or other infectious agents during an NSI [7–9], which can adversely affect both personal and professional life and can restrict career opportunities due to the risk of transmission of blood-borne pathogens to patients [9–11].

In the prevaccination era, the rate of HBV infection amongst dental-HCWs was estimated to be 3–6 times higher than in the general population [12]. Although rates amongst dental-HCWs have fallen in developed countries, in many low- and middle-income countries, vaccine coverage rates remain low and awareness of postexposure prophylaxis (PEP) is poor [13, 14]. The existing evidence base highlights that dental-HCWs appear to be at particularly high risk of NSIs [15–17]. This is mainly due to the use of sharp dental instruments often for multiple injections in the mouth where access and visibility can be poor [9, 18–20].

It is difficult to accurately estimate the global prevalence of NSIs among dental-HCWs due to the underreporting of incidents which is a significant issue in developing countries [21, 22]. Iranian studies have shown that, in some settings, over 80% of dental-HCWs fail to report NSIs [23, 24]. A national community survey which was carried out in 2007-2008 calculated that the prevalence of hepatitis B surface antigen (HBsAg) and hepatitis C virus in Pakistan were 2.5% and 4.8%, respectively, and estimated that there were approximately 13 million chronic hepatitis B and C carriers in the country [25], but this is now outdated. Taking into consideration the evidence on underreporting of NSIs, this figure could potentially be much higher, indicating dental-HCWs in Pakistan are at a particularly high risk of infection following an NSI.

A number of factors for the underreporting of NSIs are presented in the literature and include lack of awareness that NSIs need to be reported [23, 24], lack of awareness of where to report [26, 27], the belief that there is no point in reporting incidents, and unwillingness to report the incident [26]. The fear of getting blamed was also found to be a common reason among dental students [28]. There is, however, a dearth of information on the prevalence, risk factors, and reasons for underreporting NSIs among dental-HCWs in Pakistan despite the high NSI prevalence [17]. Synthesizing existing evidence on the prevalence and risk factors of NSIs and the rate and reasons of underreporting of NSIs among dental-HCWs in Pakistan can potentially underline the existing gaps in the available literature and dental practices that may require further consideration.

## 2. Aim and Objectives

The aim of this paper is to review the existing literature to determine the prevalence and rate of reporting of NSIs among dental-HCWs in Pakistan.

## 3. Methodology

### 3.1. Selection Criteria

Inclusion criteria for relevant studies were as follows:

1. Primary research studies published in peer-reviewed journals
2. Studies from Pakistan that sampled dental-HCWs
3. Studies that reported the prevalence and/or reporting rates of NSIs
4. Studies published in English between January 2000 and June 2016

## 4. Search Strategy

The search strategy included electronic database search and hand searching up to 30 June 2016. The electronic databases MEDLINE, Google Scholar, Discover, Cochrane Library, CINAHL, BMC, ScienceDirect, Web of Science, and the Directory of Open Access Journals (DOAJ) were searched using the following key words and Boolean operators: (prevalen^∗^ OR occur^∗^ OR rate^∗^ OR frequency^∗^ OR report^∗^ OR record^∗^) AND (needle^∗^ OR occupation^∗^ OR sharp^∗^ OR percutaneous) AND (injury^∗^ OR trauma^∗^ OR wound^∗^) AND (dental worker^∗^ OR dental student^∗^ OR dental assistant^∗^ OR dentist^∗^ OR dental staff) AND (Pakistan^∗^ OR South Asia^∗^ OR developing country^∗^). The titles and abstracts of the papers identified were screened against the inclusion and exclusion criteria. Additional papers were identified from searching Pakistan-based dental journals not indexed in the databases listed above, a citation search of key authors, and screening the reference lists of the papers which passed the screening test for related articles.

## 5. Data Extraction

Relevant data were extracted from the studies based on the “STROBE” framework criteria for cross-sectional studies [29]. Data were extracted and entered on a Microsoft Excel spreadsheet. The data extraction headings were as follows: author(s), year of publication, journal title, article title, study aim and objectives, study design, participants, study location, sampling technique, study size, data collection method, response rate, descriptive data, data analysis, key results, and conclusions.

## 6. Quality Appraisal

Following data extraction, the methodological quality and rigour of the included studies were assessed using Boyle's [30] quality assessment framework criteria to evaluate the potential strength of the outcomes. The quality assessment followed a scoring system comprising eight questions, and studies were graded high (7-8 score), moderate (4–6 score), or low (1–3 score) quality based on three main criteria: sampling, measurement, and analysis [30–32]. The sampling framework was applied to all selected studies in a consistent fashion, and the minimum response rate in the reviewed studies was set at 80% [30].

## 7. Data Analysis

The results were analysed using narrative analysis. A textual approach was used to combine and summarise the findings from different studies and subsequently explain the synthesised findings [33]. It was selected as it systematically evaluates and incorporates the results from across the studies and explores the similarities and dissimilarities between the study findings [34]. Since the included studies demonstrated heterogeneity with regard to their evaluation criteria and study results, performing a meta-analysis was not considered appropriate, as it would have yielded potentially insignificant and misleading results [35]. Furthermore, the data required for performing a meta-analysis were absent in all the reviewed studies [36, 37].

## 8. Methods of the Review

A review of the abstracts and titles was carried out by all the authors to determine the suitability of the papers and resolve any differences as to whether to include or exclude papers. Mehak Parveiz extracted the data and assessed quality of the data, and Ruth Gilbert and Nasreen Ali cross-checked the extracted data and quality assessment to ensure data accuracy.

## 9. Results

### 9.1. Overall Description of the Included and Excluded Studies

**Figure 1:**
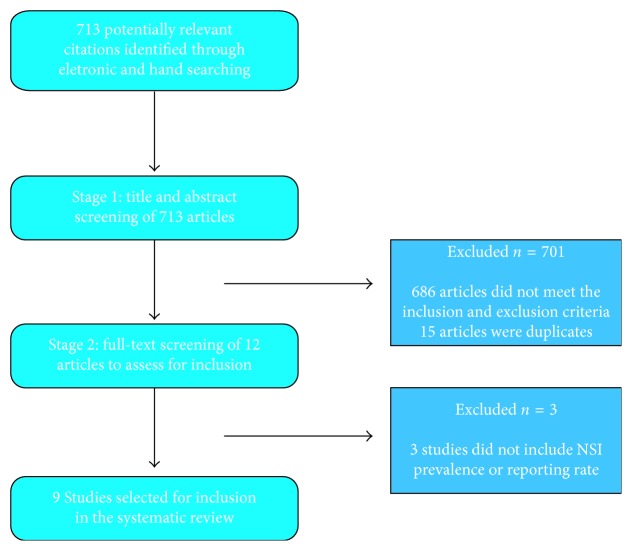
Flowchart of search strategy.

A total of 713 potentially relevant citations were identified by electronic and hand searching. Following initial screening of titles and abstracts, 15 duplicate papers were excluded and 686 studies were excluded based on the prespecified inclusion and exclusion criteria. The full-text of the remaining 12 studies was scrutinized to determine their eligibility for inclusion in the review. Of these, three further articles were excluded as they failed to mention the prevalence or reporting rates of NSIs. As a result, nine primary studies met the inclusion criteria and were included in the review (Figure 1).

### 9.2. Analysis of Included Studies

#### 9.2.1. Study Design

The nine included studies were conducted in seven different Pakistani cities: Karachi [38, 39], Hyderabad [17, 26], Lahore [40], Jamshoro [41], Quetta [42], Peshawar, and Abbottabad [43, 44]. All the studies had an observational, cross-sectional study design, which quantitatively measured the prevalence of NSIs, whereas only four studies [17, 26, 39, 41] measured the reporting rate of NSIs. All included studies were within the review's inclusion criteria as they were Pakistan-based primary studies reporting the prevalence and/or reporting rate of NSIs among dental-HCWs published between 2009 and 2015 in a peer-reviewed journal in English.

#### 9.2.2. Study Sampling

The study sample sizes ranged from 100 to 800. However, the included studies failed to specify the employed sampling technique, except for Khan et al. [43], which adopted a convenience sampling technique, though no rationale was provided. All studies used questionnaires as their measuring tool.

#### 9.2.3. Response Rate

The response rate ranged from as high as 100% [38–40] to as low as 75% [44]. However, three studies failed to take account of their response rate [17, 42, 43].

#### 9.2.4. Study Population

The gender ratio of the participants was not mentioned in three of the studies [38, 43, 44]. Nonetheless, in other studies [17, 39–42,], on average 53% of the sample were male and 47% were female, making the ratio roughly equal in all studies except for Jan et al. [26], in which 83% of the study participants were male. Almost all studies included dental-HCWs from different job categories including dentists, dental faculty, postgraduates, house officers, undergraduates, assistants, technicians, and paradental staff. However, one study [44] sampled only dentists.

#### 9.2.5. Age Range of Participants

Age of the participants was recorded by only three of the included studies. In two of the studies [39, 42], the majority of the study participants were between 20 and 30 years, whereas in one study [26], 50 participants were 25–35 years old, 73 were 36–45 years old, and 131 were older than 45 years. Six of the reviewed studies failed to report any information on the age of the participants [17, 38, 40, 41, 43, 44].

#### 9.2.6. Survey Duration

The survey duration was stated by five studies and varied considerably. Survey durations were one month [26], four months [43], nine months [38], and over one year [17, 41]. Four of the selected studies failed to take account of their study period [39, 40, 42, 44]. A full summary of the background information, methodological details, and key findings of the included studies is presented in Table 1.

## 10. Data Analysis

There were significant variations in the reporting of data on NSI prevalence, rate of reporting, and risk factors, as well as in the data on knowledge and awareness regarding NSIs and dental practices to prevent NSIs. As a result of which it was challenging to compare data across the studies.

### 10.1. Prevalence of NSIs

The prevalence of NSIs among Pakistani dental-HCWs ranged from 30% [39, 44] to 73% [38] (Table 1). In studies which compared the prevalence rate amongst different groups of dental-HCWs, dental undergraduate students generally experienced the highest rates of NSIs (15–60%) [17, 39, 41], while a lower prevalence of NSIs was observed among the qualified dentists, including dental surgeons, postgraduates, and house officers [17, 39, 41, 42] However, there were variations in the findings; Khan et al. [43] reported almost equal prevalence of NSIs amongst dentists and dental students, while Ikram et al. [38] observed that the majority (42%) of those reporting NSIs were dental house officers. All studies which included dental assistants and technicians showed that they were the group with the lowest rates of NSIs [17, 39, 41], except for one study [26] which reported that 51% of dental technicians had experienced an NSI.

Five studies recorded the number of NSIs experienced by each participant (Table 2). Baig et al. [41] and Gichki et al. [42] recorded that most dental-HCWs who had experienced an NSI experienced just one incident (64%). However, Shahzad et al. [17] and Jan et al. [26] recorded that most dental-HCWs had experienced more than one NSI (67% and 88%, resp.). Furthermore, many participants reported having experienced more than two NSIs [17, 26, 41] with 9% of participants in one study [44] reporting that they experienced more than 10 incidents during their dental career.

### 10.2. Reporting of NSIs

Only four studies asked participants whether they would report an NSI [17, 26, 38, 41]. Baig et al. [41] recorded the highest underreporting rate (76%); most participants stated that they were unaware of the reporting system. Jan et al. [26] found that 60% of dentists and 92% of dental technicians failed to report injuries. The most common reason for underreporting amongst dentists was the belief that there was no point in reporting incidents (33%), whereas amongst dental technicians, it was not knowing where the incident should be reported to, or an unwillingness to report as they were practicing illegally (59%). Shahzad et al. [17] found that 15% of NSIs were not reported, usually because those affected did not know who to report the incident to. Conversely, Malik et al. [39] noted that 28 of the 30 (93%) dental-HCWs who experienced an NSI reported it, thus making it the highest reporting rate observed amongst the included studies.

### 10.3. Risk Factors for NSIs

A number of different dental procedures appear to put dental-HCWs at risk of sustaining an NSI. NSIs most frequently occurred during needle recapping (33%) [40, 41]. Surgical procedures (28%), drawing blood samples (26%), needle exchange (17%), local anaesthesia administration (9%), and sharps disposal (12%) procedures were also responsible for many NSIs [40, 41]. Jan et al. [26] found that NSIs were most likely to occur during infiltration anaesthesia (43%), followed by general dental procedures (23%) and needle recapping (16%). Malik et al. [39], however, reported that NSIs were most likely to occur whilst disposing of gloves (94%); due to bending needles (92%); or whilst recapping (88%), discarding (69%), separating (64%); or disassembling needles (28%). Meanwhile, Shahzad et al. [17] found that infiltration anaesthesia was responsible for 55% of NSIs and block anaesthesia was responsible for 45%; no other dental procedures were reported to be associated with NSIs.

Only Shahzad et al. [17] investigated which departments had the highest rates of NSIs. The highest prevalence occurred in the oral surgery department (58%), followed by the operative department (18%), while the departments of prosthodontics, orthodontics, and periodontology had the lowest prevalence of NSIs (3% each).

Three studies investigated human factors which may have led to NSIs. Each study reported different factors. Shahzad et al. [17] reported that working hastily was the most common reason for an NSI (42%), followed by fatigue (20%), lack of skill (14%), not wearing gloves (12%), lack of supervision (5%), and the practice of needle resheathing (5%). Baig et al. [41] reported stress as the most common cause of an NSI (43%), followed by work overload (38%), carelessness (8%), and unskilled handling of the instruments (5%), whereas Gichki et al. [42] recorded that negligence among dental-HCWs was the most likely cause of an NSI (20%).

### 10.4. Hepatitis B Vaccine Coverage

Seven of the reviewed studies calculated HBV vaccine coverage rates among dental-HCWs [17, 26, 38, 40–42, 44]. Rates of vaccine coverage ranged from 46 to 93%. Baig et al. [41] and Ikram et al. [38] reported the highest coverage rates (92% and 93%, resp.). Gichki et al. [42], Ashfaq et al. [40], Mehboob et al. [44], and Shahzad et al. [17] reported vaccine coverage rates of 88%, 87%, 82%, and 68%, respectively. The lowest vaccine coverage rates (57%) were reported by Jan et al. [26]; however, 81% of dentists had received at least one dose of vaccine compared to just 10% of dental technicians.

### 10.5. Knowledge and Awareness regarding NSIs

Five studies collected information on the awareness of measures to prevent NSIs among dental-HCWs [17, 38–40, 42]. Ikram et al. [38] found that 82% of dental-HCWs had received training regarding the risk of blood-borne infections; 54% felt that training and education were important measures in preventing NSI, and 41% felt that outpatient departments (OPDs) needed to develop specific protocols to protect workers.

Malik et al. [39] reported good knowledge among dental-HCWs regarding wearing gloves (97%) and universal precautions (74%); however, 88% of participants reported that needles should be recapped or bent needles after use, and only 53% were aware of needle-less safety devices. Ashfaq et al. [40] also found that many dental workers reported they were aware of precautionary measures which could prevent NSIs and transmission of infection (85%). However, Ikram et al. [38] found that only 39% of participants agreed that using surgical gloves would reduce the risk of NSIs, and less than 5% of participants agreed that needles should not be recapped after use. When questioned about strategies to prevent NSIs, only 38% of participants suggested that needles should not be resheathed, 34% suggested that needle approximation should be done carefully, and 27% suggested using sharps containers.

Knowledge and awareness also varied between different groups of dental-HCWs. Jan et al. [26] found 63% of dentists, but only 8% of dental technicians were aware of measures which could be taken to reduce the risk of NSIs, while Gichki et al. [42] found that 76% of house officers and 63% of students were aware needles should not be recapped. Malik et al. [39] found that 98% of dental-HCWs were aware hepatitis B could be transmitted during an NSI, while only 84% were aware HCV and HIV could be transmitted in this way. Similarly, although Gichki et al. [42] reported that 98% of dental-HCWs were aware blood-borne viruses could be transmitted during an NSI, only 13% were aware that HIV could be transmitted during an NSI.

### 10.6. Dental Practices to Prevent NSIs

Dental practices used to prevent NSIs were also reviewed. One of the main precautions used to prevent an NSI was wearing of gloves; however, there was a wide variation in the proportion of dental-HCWs who reported wearing gloves. Malik et al. [39] found that over 90% of dental-HCWs reported wearing gloves during phlebotomy, while withdrawing a needle from a patient, disposing of the contaminated needle, and when manipulating the sharps bin. Gichki et al. [42] found that 73% of dental-HCWs wore gloves; however, practice varied between students and qualified dental-HCWs (69% of students and 83% of house officers). Similarly, Khan et al. [43] recorded variation in practice between different groups of dental-HCWs (68% of all dental-HCWs wore gloves, 86% of students, and 44% of qualified dentists). Khan et al. [43] also reported that 79% of respondents would change their gloves if they became dirty during a procedure. Some studies noted that other personal protective equipment was used. Mehboob et al. [44] found that 86% of dental-HCWs used masks and gloves as precautionary measures, but only 9% of dentists used all the recommended universal precautions during dental treatment. Meanwhile, Khan et al. [43] found that 10% of dental-HCWs wore goggles and 90% wore facemasks.

Several studies identified safe disposal of needles as playing an essential role in preventing NSIs. Khan et al. [43] noted that 65% of dental-HCWs reported they disposed of needles safely (60% of qualified dental-HCWs and 68% of students); however, only 16% (23% of qualified dental-HCWs and 11% of students) avoided resheathing needles after injecting local anaesthetic. Similarly, Malik et al. [39] found that only 12% of dental-HCWs avoided recapping needles after use, and approximately a third (36%) avoided separating the needle and syringe before disposal. By contrast, the more recent study by Gichki et al. [40] found that 67% of dental-HCWs did not recap needles after use (63% students and 76% house officers).

## 11. Discussion

Nine studies were identified which reported data on the prevalence and reporting rates of NSIs amongst dental-HCWs in Pakistan. In each study, the prevalence of NSIs among dental-HCWs in Pakistan was found to be high, ranging from 30% [39] to 73% [38]. The findings were consistent with previous studies from other low- and middle-income countries, including Thailand, Colombia, Saudi Arabia, Iran, Romania, Nigeria, Jordan, and China [13, 22, 45–50]. They also confirmed that dental-HCWs in Pakistan were more likely to experience an NSI than dental-HCWs in developed countries. Only 14% of dentists in Scotland reported that they had experienced an NSI [51], while in UAE, Taiwan, and Australia, approximately 25% of dental-HCWs reported that they had experienced an NSI [10, 52, 53].

From the review, it was evident that many dental-HCWs in Pakistan experience multiple NSIs. Although Baig et al. [41] and Gichki et al. [42] found that most dental-HCWs who had experienced an NSI experienced just one incident (64%), indicating that the incident led to a change in practice; in other studies, many dental-HCWs reported that they had experienced multiple injuries. Consequently, NSIs represent a serious health and safety concern for dental-HCWs. Similarly, other studies conducted in low- and middle-income countries have concluded that over half of dental-HCWs have been exposed to more than one NSI [22, 23, 54]. Furthermore, Jan et al. [26] reported that participants had experienced multiple NSIs in the preceding 12 months, indicating that NSIs remain an ongoing, contemporary risk to dental-HCWs. These findings highlight the need to investigate differences in policies and working practices to identify how rates of NSIs can be effectively reduced in Pakistan and other countries with high rates of injury. Experience appears to be one of a number of factors which play an important role in reducing rates of NSIs. Dental undergraduate students appeared to be more likely to experience an NSI than experienced, qualified dentists. Similarly, the youngest dental-HCWs with the least experience were found to encounter more NSIs than older practitioners with more years of experience [41]. Presumably, this is in part due to a lack of experience when starting clinical practice; however, the heavy clinical load allocated during dental training was also reported to be a reason behind the high rates of NSI among dental students [17]. Dental assistants and technicians reported the lowest rate of NSIs, possibly due to having been in practice longer and having more experience than the other groups of dental-HCWs [17, 39, 41]. However, data from this group were limited, and consequently, it is difficult to draw firm conclusions. These findings were consistent with studies from many other countries, which also reported that dental students were the group most likely to experience an NSI due to their limited experience, skills, and frequent use of sharp instruments [4, 19, 54, 55]. Interestingly, in some settings, experienced or older dental-HCWs were found to be more likely to experience NSIs; in these cases, workload was cited as a key risk factor [52, 56, 57]. Consequently, limited clinical skills, knowledge and experience, and workload all appear to increase the risk of NSIs for dental-HCWs. The evidence highlights the need to review the clinical workload of all dental-HCWs, to prevent work overload, stress, and fatigue, as well as the provision of adequate training and mentoring to reduce the risk of NSIs.

However, data on NSI prevalence are limited in Pakistan, and more robust surveillance data would help to support effective policy development. These studies confirmed that although most NSIs are officially reported in some settings [39, 42], underreporting of NSIs is an ongoing problem in Pakistan with over 75% of NSIs not being reported in some settings [41]. The problem appears to stem from many dental-HCWs being unaware of the reporting system and failing to understand the importance of reporting incidents [17, 41]. Furthermore, some groups of dental-HCWs, such as dental technicians, appear to be particularly reluctant to report NSIs [26]. Poor surveillance of NSIs appears to be a widespread issue. Reporting rates in Pakistan were broadly in-line with rates in other low- and middle-income nations. Studies have shown that more than half of dental-HCWs failed to report their NSIs in Saudi Arabia, Kenya, and India [22, 23, 57] and more than three-quarters of dental-HCWs failed to report NSIs in China, North Jordan, and Iran [22, 23, 57]. Furthermore, in Nigeria, a study from one dental setting found that none of the dental students reported NSIs [27]. Similarly, reasons for underreporting of NSIs included fear of the consequences of infection, stigmatisation and blame, lack of awareness of the need to report NSIs, and not knowing how or where to report an NSI [23, 24, 27]. These findings highlight a widespread lack of awareness regarding reporting NSIs and indicate the need for further training and guidance to improve reporting rates and strengthen reporting systems.

The included studies also provided insight into which working practices were most likely to result in an NSI. The results revealed that needle recapping or resheathing was the procedure responsible for the greatest number of NSIs [40, 41]. Furthermore, bending a needle prior to disposing it also appeared to be a risk-prone procedure [39]. Similar findings have been reported from other low- and middle-income countries including Iran, India, and China [28, 49, 57]. Despite WHO [50] recommendations that all HCWs should avoid recapping needles or bending, breaking, or manually removing needles before disposal, the majority of dentists in some settings still report resheathing needles [38, 43]. Consequently, to effectively reduce the risk of NSIs, it is essential that working policies and practices are updated to encompass the latest best practice. However, even if policies and protocols are based on best practice guidance, many factors will affect rates of compliance. An individual's practice can be determined by the behavioural theory of health-belief model [58]. Analysis showed that in some settings, a high proportion of dental-HCWs was aware of good practice, such as wearing gloves, safe needle practice, and improved engineering-controlled devices [39, 40, 42–44], whereas it was found to be low in other settings [38]. Likewise, perception of the risk of transmission of infection was found to vary considerably between settings [39, 42], as were hepatitis B vaccine coverage rates [17, 26, 38, 40–42, 44] and understanding of PEP [38, 41, 42]. Consequently, practice and perceived susceptibility which potentially influences decisions to observe precautions was found to be variable between settings. The high prevalence of NSIs, particularly among dental students, indicates a crucial need for dental-HCWs to understand the risk of NSI-associated infections, in order for them to appreciate the importance of complying with the universal precautions and other safe working procedures. Thus, it is essential that education on NSI risks and prevention strategies is included early in the dental course curriculum and repeated regularly as part of ongoing continual professional development (CPD).

The review also highlighted that hepatitis B vaccine coverage was extremely variable both between settings and different groups of dental-HCWs. Therefore, measures should be put in place to ensure that all dental-HCWs have access to affordable hepatitis B immunisation and good coverage rates are achieved amongst all groups of dental professionals. However, since there are no effective vaccines available to protect against HCV and HIV infection, and their treatment is neither affordable nor available in many countries, it is essential that dental-HCWs continue to be aware of the importance of developing good practice to avoid NSIs.

To the best of the authors' knowledge, this is the first systematic review of its kind to highlight the issue of NSIs in dental-HCWs in Pakistan. In absence of the routine collection of accurate data on NSIs, small studies have been useful in highlighting which groups of dental-HCWs are most at risk from NSIs. A major limitation of this systematic review was the low quality of the reviewed studies, thus raising serious quality concerns for the review, which impacts the reliability, credibility, and applicability of the overall results, and consequently the drawn conclusion and recommendations of the review [36]. However, the quality assessment outcome recommends the need for further good-quality studies with robust methodology to increase the transparency, validity, and generalisation of the research outcomes and also highlights gaps in the present literature. Despite these limitations, it can be concluded that a high prevalence of NSIs and low rates of reporting, as well as a lack of awareness of the risks of NSIs, persist in many settings within Pakistan.

## 12. Conclusion

Reviews of the selected studies suggest that the prevalence of NSIs among dental-HCWs in Pakistan is high while reporting rates are low, suggesting the urgent need to develop educational programmes for all dental-HCWs on the importance of preventing and reporting NSIs. It also indicates the necessity for all dental-HCWs to be able to access a proper occupational health department in all dental settings, to prevent, manage, record, and monitor occupational injuries. There is an urgent need for the development of national guidance protocols to prevent NSIs in Pakistan. Improving health literacy around the risks of NSIs should be accompanied by improving measures to report NSIs. These should incorporate examples of good practice from countries where rates of NSIs have successfully been reduced. However, it is important to note that recommendations for new interventions should take an ecological approach and should be cost-effective for the dental settings since this is crucial for their successful and sustainable application.

## Figures and Tables

**Table 1:** Summary of the included studies.

Serial number	Authors and year of publication	Study location	Research aim and objectives	Study design and time frame	Sample, recruitment method, participant details, characteristics, and response rate	Study outcome measures	Key results	Quality ranking
(1)	Ashfaq et al. (2011) [40]	Lahore Medical and Dental College, Lahore, Pakistan.	To assess the degree of awareness of NSIs among dental health professionals.	Cross-sectional study, questionnaire survey in 2010.	In total, 139 dental health professionals including dental students (*n* = 55), graduates (*n* = 63), and staff (*n* = 21) were sampled (sampling technique was not specified). Participants were of mixed gender: *n* = 76 males and *n* = 63 females. The response rate was 100%.	Prevalence and frequency of NSI exposures, mechanism of the injury, knowledge of precautionary measures to prevent NSIs, knowledge of first aid management of NSIs, and participant's HBV vaccination status.	In total, 45% (*n* = 63) of dental health professionals were exposed to NSIs. NSIs resulted from needle recapping (*n* = 33), needle exchange (*n* = 17), sharps disposal, (*n* = 12) and local anaesthetic administration (*n* = 9). A large number of participants were aware of precautionary measures (*n* = 132) and first aid management (*n* = 118). The HBV vaccine coverage was also high (*n* = 121).	Low
(2)	Baig et al. (2014) [41]	Maxillofacial Surgery Department, Liaquat University of Medical and Health Sciences, Jamshoro, Pakistan.	To estimate the risk of NSIs, their frequency, nature, and awareness level of prophylaxis among the students, house officers, and supporting staff of dentistry.	Descriptive cross-sectional study, questionnaire survey from April 2012 to April 2013.	In total, *n* = 613 individuals including maxillofacial surgeons (30%, *n* = 181), general dentists/supporting staff (3%, *n* = 21), house officers (18%, *n* = 107) and undergraduate students (49%, *n* = 298) were sampled (sampling technique was not stated). The sampled participants were of mixed gender comprising 48% (*n* = 289) males and 52% (*n* = 318) females. The response rate was 99%.	Prevalence, frequency, and predisposing factors of NSIs, awareness of PEP, and HBV vaccination coverage of dental-HCWs.	A high number of NSIs (*n* = 776) were experienced by 60% (*n* = 363) of the participants. Of those who experienced an NSI, 40% were students, 38% were dental surgeons, 12% were house officers, and 4% were supporting staff. 64% (*n* = 233) of dental-HCWs experienced one NSI, 18% (*n* = 64) experienced two NSIs, 12% (*n* = 42) experienced three NSIs, and 7% (*n* = 26) experienced more than three. NSIs resulted from needle recapping 33% (*n* = 87), surgical procedures 28% (*n* = 73), or drawing blood samples 26% (*n* = 69). The most common reasons behind NSIs among the participants were stress 43% (*n* = 112), work overload 38% (*n* = 99), carelessness 8% (*n* = 30), and unskilled instrument handling 5% (*n* = 17). Almost 70% reported NSIs as self-injuries. Overall, almost 76% of NSIs were unreported by dental-HCWs, due to lack of awareness of the reporting system. HBV vaccine coverage was 92%.	Low
(3)	Gichki et al. (2015) [42]	Dental section, Bolan Medical College, Sandeman Provincial Hospital, Quetta, Pakistan.	To assess the knowledge and awareness of NSIs among house officers and dental students.	Observational cross-sectional study, self-administered questionnaire survey. Study period not mentioned.	A total of 100 participants including house officers (29%) and dental students (71%) participated. The age range of the participants was 21–29 years; they were of mixed gender with 44% males and 56% females. The sample recruitment technique as well as the response rate was not mentioned.	Prevalence, frequency, and predisposing factors of NSIs. Knowledge and awareness of the transmission of BBV, availability of vaccines, importance of reporting NSIs, and initiating PEP. Participant's precautionary measures towards NSIs and vaccination status.	A total of 33% of participants experienced an NSI; 21% experienced one NSI and 12% experienced more than one. Good knowledge of the following aspects was reported: NSIs (97%); risk of transmission of BBVs (98%); transmission of pathogens (71%); availability of HBV vaccine (83%); preventing NSIs through needle recapping (69%) and engineering control devices (84%); reporting NSIs (99%); needle recapping technique (61%); and different stages of PEP (60–91%). Knowledge of the risk of HIV transmission through NSIs was the weakest area (13%) and 20% admitted their NSI was due to negligence. 73% of the respondents wore gloves while practicing, 67% avoided the practice of needle recapping. 88% were vaccinated against HBV.	Low
(4)	Ikram et al. (2015) [38]	8 different institutes of Karachi, Pakistan.	To assess the frequency of NSIs and knowledge, attitude, and practice of dental workers towards NSI prevention.	Descriptive cross-sectional study, questionnaire survey from July 2014 to March 2015.	A sample of 800 participants, comprising undergraduates (23%), house officers (42%), faculty (25%), and general dental practitioners (10%), were included. Sampling technique was not stated. The response rate was 100%.	Prevalence of NSIs and the knowledge, attitude, and practices related to NSIs.	In total, 73.1% of participants gave a history of NSI during their dental practice. 92.8% had received HBV vaccine and 73% believed in the effectiveness of the vaccination, whereas only 38.5% believed in the effectiveness of gloves in reducing the occurrence of NSIs. Needle recapping was practiced by 51% of the participants, but 38.3% suggested that it should be avoided, whereas 34.4% and 27.4% suggested careful needle approximation and use of sharps containers, respectively. 70.1% were aware of PEP after an NSI and 82.3% agreed that they were provided with instructions about the risk of infections in their training, but 54.4% suggested the need for training and education, and 40.5% suggested revising protocols in outpatient departments OPDs.	Low
(5)	Jan et al. (2014) [26]	Independent private dental clinics in Hyderabad and Karachi, Pakistan.	To determine the frequency of NSIs among dental-HCWs including dental technicians.	Cross-sectional study, self-administered questionnaire survey during April 2013.	A total of 254 dental-HCWs including qualified dentists (*n* = 166) and dental technicians (*n* = 88) were selected, with no mention of the sampling strategy. The participants were of mixed gender: males (*n* = 209) and females (*n* = 45); from different age groups: 25–35 years old (*n* = 50), 36–45 years old (*n* = 73) and over 45 years old (*n* = 131). The response rate was 92.3%.	Prevalence and frequency of NSIs during the previous year. Reporting rates of NSIs and reasons for underreporting. Also, the knowledge and attitude towards universal precautions.	53% (135) of the 254 participants (qualified dentists and dental technicians) had experienced at least one NSI in the preceding 12 months. Among dentists, 54% experienced at least one NSI; 35% experienced two; and 11% experienced more than two NSIs. Among dental technicians, 51% experienced at least one NSI; 28% experience two; and 21% more than two. Infiltration anaesthesia was the most common procedure causing NSIs (44.4% among dentists and 42% among dental technicians). 59.6% of dentists did not report their NSI; the most common reason given was lack of belief in the reporting system (33.1%), whereas 92% of dental technicians did not report their NSI; the most common reason given was not knowing where to report or did not want to report (59.1%). Dentists (62.6%) had more knowledge about the safety guidelines than dental technicians (8%) and also had a better vaccination coverage (81.3%) than dental technicians (10.2%).	Low
(6)	Khan et al. (2009) [43]	Sardar Begam Dental College, Peshawar, Pakistan.	To evaluate the perception of cross infection in dental practice among dental surgeons and dental students	Descriptive cross-sectional study, survey questionnaire from December 2007 to March 2008.	Total 100 dental-HCWs including 43% dentists (consultants, demonstrators, and house officers) and 57% undergraduate students were sampled through convenience sampling technique. The demographic characteristics and response rate were not mentioned.	Prevalence of NSI, and knowledge and practice of infection control measures.	A total of 35% dental health professionals experienced an NSI during their dental career. The majority of them washed and covered it after allowing it to bleed (85%). Most of the participants also took the patient's medical history (79%) and screened the patient (65%). 65% of dental workers practiced safe disposal, whereas 84% practiced needle resheathing after administering injection, in which one-handed technique was the most common (49%).	Low
(7)	Malik et al. (2012) [39]	Oral surgery department, Dr. Ishrat-ul-Ebad Khan Institute of Oral Health Sciences, Karachi, Pakistan.	Assess knowledge, attitude, and practices relating to NSIs and risk factors among dental practitioners.	Cross-sectional study, survey questionnaire; study period not mentioned.	A total of 100 participants including undergraduates (62%), postgraduates (21%), graduates (13%), and staff (4%) participated, with no identified strategy for sampling. They were of mixed gender: 55% females and 45% males. The majority (85%) were 20–30 years and most (94%) had 1–5 years' experience. The response rate was 100%.	Knowledge, attitude, and practice regarding NSIs, including prevalence and reporting rates.	Of the total 100 participants, 30 experienced an NSI, of which 28 were reported. NSIs were highest among dental students, age group 20–30 years, and practitioners with 1–5 years' experience. The participants were aware of the universal precautions (74%), hepatitis B (98%), hepatitis C, and HIV/AIDS transmission (84%) via NSIs, whereas only 53% were aware of needle-less safety devices. Needle recapping was practiced by 88% and was reported as one of the common reasons for NSIs (88%), along with disposing of gloves (94%). A large number of the participants wore gloves when disposing needles (94%) and manipulating the sharps bin (92%).	Moderate
(8)	Mehboob et al. (2012) [44]	Two teaching hospitals: Khyber College of Dentistry Peshawar, Pakistan, and Ayub Medical College, Abbottabad, Pakistan.	To determine the prevalence and awareness of professional hazards including psychological, musculoskeletal, biological, and allergic problems among dentists.	Cross-sectional study, survey questionnaire-study period not mentioned.	A total of 113 dentists were sampled. The sampling strategy was not specified. The participants included dental graduates (50%) and postgraduate trainees (35%), and the rest were the members or fellows of the college of physicians and surgeons. The majority of the participants (61%) had work experience of less than 5 years. Out of 150, 37 questionnaires were not returned (75% response rate).	The prevalence and frequency of NSIs and the precautions used by dentists during dental treatment.	70% of the participants were exposed to NSIs; 54% experienced less than 5 NSIs; 9.7% experienced 5–10 NSIs; 6.2% had more than 10 NSIs. Only 8.8% of the participants used all precautions during treatment, whereas the majority (85.8%) used a combination of 2 precautions, usually including gloves and masks. 82.3% of participants were vaccinated against HBV.	Low
(9)	Shahzad et al. (2013) [17]	Liaquat Medical University Hospital, Hyderabad, Pakistan.	To identify the risks of NSIs, the participants who sustained them, the circumstances under which they occurred, and how the risk of NSI was minimised among the participants.	Descriptive cross-sectional study, survey questionnaire-from August 2011 to September 2012.	In total, 513 participants including dental students (*n* = 325), house officers (*n* = 80), and paradental staff (*n* = 108), were included in the study. The sampling strategy was not specified. They were of mixed gender: 58% females and 42% males. The response rate was not taken into consideration.	Frequency and reporting rates of NSIs. The type of technique which caused NSIs, the department-wide distribution of NSIs, the different reasons of NSIs, and vaccination status of the participants.	773 total injuries occurred among the participants; 52% were students; 21% were dentists in their first professional year; 10% were paradental staff. 15% of all injuries went unreported. The NSI prevalence was the highest in the oral surgery department (58%), followed by the operative department (18%), and was the lowest in prosthodontics, orthodontics, and periodontology departments (3% each). NSIs most frequently occurred during infiltration anaesthesia (55%), followed by block anaesthesia (44%). The most common reasons for an NSI were hurrying (42%), fatigue (20%), lack of skill (14%), and not wearing gloves (12%). HBV vaccination coverage was 68%.	Low

**Table 2:** Frequency of needlestick injuries amongst dental-HCWs who reported they had experienced at least one NSI.

Study	Number of needlestick injuries				
	1 NSI	>1 NSI	>2 NSIs	<5 NSIs	≥5 NSIs
Baig et al. (2014) [41]	233 (64%)	132 (36%)	68 (19%)	NA	NA
Gichki et al. (2015) [42]	21 (64%)	12 (36%)	NA	NA	NA
Jan et al. (2014)^∗^ [26]	16 (12%)	119 (88%)	36 (27%)	NA	NA
Mehboob et al. (2012) [44]	NA	NA	NA	61 (77%)	18 (23%)
Shahzad et al. (2013) [17]	89 (33%)	179 (67%)	121 (45%)	NA	NA

^∗^NSIs were reported in preceding 12 months. NA = not available.

## References

[1] World Health Organization–WHO (2016). Occupational health, needlestick injuries. http://www.who.int/occupational_health/topics/needinjuries/en/.

[2] National Health Services–NHS (2015). What should I do if I injure myself with a used needle?. http://www.nhs.uk/chq/Pages/2557.aspx?CategoryID=72.

[3] Smith A., Cameron S., Bagg J., Kennedy D. (2001). Management of needlestick injuries in general dental practice. *British Dental Journal*.

[4] Gatto M., Bandini L., Montevecchi M., Checchi L. (2013). Occupational exposure to blood and body fluids in a department of oral sciences: results of a thirteen-year surveillance study. *The Scientific World Journal*.

[5] Canadian Centre for Occupational Health and Safety-CCOHS (2015). Needlestick and sharps injuries. http://www.ccohs.ca/oshanswers/diseases/needlestick_injuries.html.

[6] Naghavi S. H., Shabestari O., Alcolado J. (2013). Post-traumatic stress disorder in trainee doctors with previous needlestick injuries. *Occupational Medicine*.

[7] Ayatollahi J., Ayatollahi F., Ardekani A. (2012). Occupational hazards to dental staff. *Dental Research Journal*.

[8] Centres for Disease Control and Prevention–CDC (2013). Infection control. http://www.cdc.gov/oralhealth/infectioncontrol/faq/bloodborne_exposures.html.

[9] Rashid H. (2014). Needle stick injuries in restorative dentistry: the need for prevention. *Journal of Restorative Dentistry*.

[10] Jaber M. (2011). A survey of needle sticks and other sharp injuries among dental undergraduate students. *International Journal of Infection Control*.

[11] Sharma R., Rasania S., Verma A., Singh S. (2010). Study of prevalence and response to needle stick injuries among health care workers in a Tertiary Care Hospital in Delhi, India. *Indian Journal of Community Medicine*.

[12] Alavian S., Mahboobi N. (2011). Hepatitis B infection in dentistry setting needs more attention. *Medical Principles and Practice*.

[13] Azodo C., Ehigiator O., Ojo M. (2010). Occupational risks and hepatitis B vaccination status of dental auxiliaries in Nigeria. *Medical Principles and Practice*.

[14] Singhal V., Bora D., Singh S. (2009). Hepatitis B in healthcare workers: Indian scenario. *Journal of Laboratory Physicians*.

[15] Kohn W., Collins A., Cleveland J., Harte J., Eklund K., Malvitz D. (2003). Guidelines for infection control in dental health-care settings. *Morbidity and Mortality Weekly Report-MMWR*.

[16] Kotelchuck D., Murphy D., Younai F. (2004). Impact of underreporting on the management of occupational bloodborne exposures in a dental teaching environment. *Journal of Dental Education*.

[17] Shahzad M., Hassan S., Memon M., Bashir U., Shams S. (2013). Needle stick injuries among dental students, house officers and paradental staff working at Liaquat Medical University Hospital, Hyderabad. *Pakistan Oral and Dental Journal*.

[18] Younai F., Murphy D., Kotelchuck D. (2001). Occupational exposures to blood in a dental teaching environment: results of a ten-year surveillance study. *Journal of Dental Education*.

[19] Ebrahimi S., Shadman N., Ghaempanah I. (2013). Needlestick injuries in dentists and their assistants in Kerman, Iran: prevalence, knowledge, and practice. *Journal of Oral Health and Oral Epidemiology*.

[20] Sawyer M. (2010). *Preventing Needle-Stick Injuries and the Use of Dental Safety Syringes*.

[21] Gambhi R., Kapoor V. (2015). Knowledge, awareness and practice regarding needle stick injuries in dental profession in India. *International Journal of Preventive Medicine*.

[22] Khader Y., Burgan S., Amarin Z. (2009). Self-reported needle stick injuries among dentists in North Jordan. *East Mediterranean Health Journal*.

[23] Askarian M., Malekmakan L. (2006). The prevalence of needle stick injuries in medical, dental, nursing and midwifery students at the university teaching hospitals of Shiraz, Iran. *Indian Journal of Medical Sciences*.

[24] Hashemipour M., Sadeghi A. (2007). Needlestick injuries among medical and dental students at the University of Kerman. A questionnaire study. *Journal of dentistry*.

[25] Qureshi H., Bile K. M., Jooma R., Alam S. E., Afridi H. U. (2010). Prevalence of hepatitis B and C viral infections in Pakistan: findings of a national survey. *East Mediterranean Health Journal*.

[26] Jan S., Akhund T., Akhtar M., Shaikh J. (2014). Needle stick injuries among dental health care providers: a survey done at Hyderabad and Karachi. *Pakistan Oral and Dental Journal*.

[27] Sofola O., Folayan M., Denloye O., Okeigbemen S. (2007). Occupational exposure to bloodborne pathogens and management of exposure incidents in Nigerian Dental Schools. *Journal of Dental Education*.

[28] Pavithran V. K., Murali R., Krishna M., Shamala A., Yalamalli M., Kumar A. (2015). Knowledge, attitude, and practice of needle stick and sharps injuries among dental professionals of Bangalore, India. *Journal of International Society of preventive and Community Dentistry*.

[29] (2009). STROBE Statement, STROBE checklists, checklist for case-control studies. http://www.strobe-statement.org/index.php?id=available-checklists.

[30] Boyle M. (1998). Guidelines for evaluating prevalence studies. *Evidence Based Mental Health*.

[31] Barreto L., Oliveira F., Nunes S. (2016). Epidemiologic study of charcot-marie-tooth disease: a systematic review. *Neuro-Epidemiology*.

[32] Etemadifar M., Nasr Z., Khalili B., Taherioun M., Vosoughi R. (2015). Epidemiology of neuromyelitis optica in the world: a systematic review and meta-analysis. *Multiple Sclerosis International*.

[33] Popay J., Roberts H., Sowden A. (2006). *Guidance on the Conduct of Narrative Synthesis in Systematic Reviews*.

[34] Ryan R. (2013). *Cochrane Consumers and Communication Review Group: Data Synthesis and Analysis*.

[35] Brugha T., Matthews R., Morgan Z., Hill T., Alonso J., Jones D. (2012). Methodology and reporting of systematic reviews and meta-analyses of observational studies in psychiatric epidemiology: systematic review. *British Journal of Psychiatry*.

[36] Boland A., Cherry G., Dickson R. (2014). *Doing a Systematic Review: a Student’s Guide*.

[37] Higgins J., Thompson S., Deeks J., Altman D. (2003). Measuring inconsistencies in meta-analysis. *British Medical Journal*.

[38] Ikram K., Siddiqui H., Maqbool S., Altaf M., Khan S. (2015). Frequency of needle stick injury among dental students and dentists in Karachi. *World Journal of Dentistry*.

[39] Malik A., Shaukat M., Qureshi A. (2012). Needle-stick injury: a rising bio-hazard. *Journal of Ayub Medical College Abbottabad*.

[40] Ashfaq M., Chatha M., Sohail A. (2011). Awareness of needlestick injuries among the dental health professionals at Lahore Medical and Dental College. *Pakistan Oral and Dental Journal*.

[41] Baig M., Baloch S., Muslim M. (2014). Estimation of risk of needle stick injury and the level of awareness of prophylaxis among the students, house officers and supporting staff of dentistry. *New York Science Journal*.

[42] Gichki A., Islam A., Murad W. (2015). Knowledge and awareness about needle stick injuries among dental students of Bolan Medical College, Quetta. *Pakistan Oral and Dental Journal*.

[43] Khan A., Rahim A., Bangash T., Chugtai M., Mehboob Z. (2009). Infection control in dentistry knowledge and practice regarding barrier techniques, post exposure management and prophylaxis–a study. *Pakistan Oral and Dental Journal*.

[44] Mehboob B., Khan M., Fahim-ud-din Khan A., Qiam F. (2012). Professional hazards among dentists of the two public sector teaching hospitals of Khyber Pakhtunkhwa province of Pakistan. *Pakistan Oral and Dental Journal*.

[45] Ansari S., Aldaijy M., Almijlad A. (2015). Determining the prevalence and awareness of needlestick injuries among dental health professionals in Riyadh, Saudi Arabia. *International Journal of Current Research*.

[46] Arrieta-Vergara K., Diaz-Cárdenas S., González-Martínez F. (2013). Prevalence of occupational accidents and related factors in students of dentistry. *Revista de Salud Publica*.

[47] Barlean L., Danila I., Saveanu I., Balcos C. (2013). Occupational health problems among dentists in Moldavian Region of Romania. *Revista medico-chirurgicala a Societatii de Medici si Naturalisti din Iasi*.

[48] Chowanadisai S., Kukiattrakoon B., Yapong B., Leggat P. (2000). Occupational health problems of dentists in Southern Thailand. *International Dental Journal*.

[49] Shaghagian S., Golkari A., Pardis S., Rezayi A. (2015). Occupational exposure of Shiraz Dental Students to patients’ blood and body fluid. *Journal of Dentistry Shiraz University of Medical Sciences*.

[50] World Health Organization–WHO (2010). *Best Practices for Injections and Related Procedures Toolkit*.

[51] Leavy P., Templeton A., Young L., McDonnell C. (2014). Reporting of occupational exposures to blood and body fluids in the primary dental care setting in Scotland: an evaluation of current practice and attitudes. *British Dental Journal*.

[52] Cheng H., Su C., Yen A., Huang C. (2012). Factors affecting occupational exposure to needlestick and sharps injuries among Dentists in Taiwan: a nationwide survey. *PLoS One*.

[53] Leggat P., Smith D. (2008). Prevalence of percutaneous exposure incidents amongst dentists in Queensland. *Australian Dental Journal*.

[54] Bindra S., Reddy K., Chakrabarty A., Chaudhary K. (2014). Awareness about needle stick injures and sharps disposal: a study conducted at Army College of Dental Sciences. *Journal of Maxillofacial and Oral Surgery*.

[55] Guruprasad Y., Chauhan D. (2011). Knowledge, attitude and practice regarding risk of HIV infection through accidental needlestick injuries among dental students of Raichur, India. *National Journal of Maxillofacial Surgery*.

[56] Martins A., Santos N., Lima M., Pereira R., Ferreira R. (2010). Needlestick and sharp instrument injuries among dentists in Montes Claros, Brazil. *Arquivos em Odontologia*.

[57] Xu Y., Zhu J., Huang C., Hu X., Xiong Y. (2013). Occupation exposure to blood and body fluids among dental personnel in a Chinese Dental Hospital. *Chinese Journal of Dental Research*.

[58] Glanz K., Rimer B., Viswanath K. (2008). *Health Behavior and Health Education, Theory, Research and Practice*.

